# The Emerging Role of Telemedicine in Managing Glycemic Control and Psychobehavioral Aspects of Pregnancy Complicated by Diabetes

**DOI:** 10.1155/2014/621384

**Published:** 2014-09-10

**Authors:** Nino Cristiano Chilelli, Maria Grazia Dalfrà, Annunziata Lapolla

**Affiliations:** Department of Medicine (DIMED), University of Padova, Via Giustiniani n 2, 35100 Padova, Italy

## Abstract

There is a gradual decline in concern of specialists who follow up the care of pregnant women with diabetes. In addition, due to the dwindling economic resources allocated to health services, access to specialized healthcare facilities is becoming more difficult. Telemedicine, or medicine practiced at a distance, is inserted in this context with applications differing for type of interaction (real-time or deferred, i.e., videoconferencing versus store-and-forward data transmission), type of monitoring (automatic versus requesting cooperation from the patient), and type of devices used (web connections and use of mobile phones or smartphones). Telemedicine can cope with the current lack of ability to ensure these patients frequent direct contact with their caregivers. This approach may have an impact not only on the classical maternal-fetal outcome, but also on some underestimated aspects of patients with diabetes in pregnancy, in this case their quality of life, the perception of “diabetes self-efficacy,” and the glycemic variability. In this paper, we will analyze the current evidence regarding the use of telemedicine in pregnancies complicated by diabetes, trying to highlight the main limitations of these studies and possible strategies to overcome them in order to improve the effectiveness of future clinical interventions with these medical applications.

## 1. Changing Epidemiological Scenarios Generate Changing Clinical Approaches: The Case of Telemedicine

The rationale behind telemedicine is to use information and communication technology (ICT) for better cooperation between patients and health care providers. Telemedicine focuses especially on the management of chronic diseases, including the task of training patients to collect information on their illness, submit it to clinical databases, and correctly interpret health professionals' feedback. In a word, its aim is to make patients independent in the management of their condition.

Telemedicine is proposed to compensate for the current scarcity of the resources needed to keep all patients in close contact with their health care provider by means of applications such as teleconsultation and videoconferencing. ICT is implemented to enable the provision of medical care at a distance. Telemedicine naturally relies on various applications, different modes of interaction, and diversified technical devices [1]. The most encouraging technology nowadays is teleconsultation, involving telemonitoring schemes that include asynchronous exchanges between patients and their healthcare providers (e.g., e-mails, text messages on mobile phones, automated messaging, or other methods requiring no face-to-face contact) or synchronous communications in the form of face-to-face contact using videoconferencing equipment (television, digital camera, webcam, and videophone) to connect healthcare providers to one or more patients at the same time, as well as for the purpose of providing education and training [2].

The idea behind these applications is to increase the quantity and quality of information exchanged between patient and health professional, ensuring that this exchange is educational and motivating for the former and practical for the latter. With this change of perspective, patients should become more involved in the management of their problems, and this should help them to make their lifestyles increasingly compatible with their chronic disease [10, 11].

The impact of telemedicine on the management and outcomes of diabetes in pregnancy, for both mother and offspring, has been only partially investigated (Tables 1 and 2). Hence the present review is to gather data and existing evidence on this topic (relating to gestational diabetes [GDM] and pregestational diabetes), to analyze the strengths and limitations of clinical studies conducted so far, and to try to finally outline the key points to aim to improve the effectiveness of future clinical interventions.

## 2. Contribution of Telemedicine in Pregnancy Complicated by Diabetes: Impact on Clinical and Nonclinical Outcomes

There is a consolidated body of evidence in the literature to support the relationship between good glycemic control and the lower incidence of congenital malformation and perinatal complications in pregnancy complicated by diabetes [12]. In parallel, there has been growing epidemiological prevalence of pregnancies in diabetic women, and the dwindling economic resources allocated to public health services have gradually come to limit the amount of attention specialists pay to the follow-up of these women. The difficulties of managing these cases are mainly attributable to logistical factors (attending a metabolic care unit may not be easy for pregnant women), but also to social and occupational issues (absences from work, since weekly visits are sometimes necessary). Hence, the growing use of alternative clinical strategies relies on advances in technology.

Using telemedicine to support pregnant women with diabetes could have an impact not only on the classical maternal-fetal outcomes, but also on other aspects not always taken into due account in the management of these patients, that is, their quality of life, their perception of the effectiveness of care “diabetes self-efficacy”, and their glycemic variability [17].

ICT can help us manage diabetic patients by providing additional clinical support, something now increasingly difficult to provide through classical face-to-face interactions due to the ever more limited health resources available.

Telemedicine applies in particular to the management of diabetes, especially in pregnancy complicated by diabetes, given the drastically reduced time available for examining and educating these patients who need short-term adjustments to their therapy and reassurance concerning an appropriate diet, as well as routine care [18]. Making the importance of an intensive approach to these patients compatible with the need to contain the cost of care is crucial to the management of diabetes in pregnancy and telemedicine can have a key role.

Studies evaluating these applications must take into account both clinical aspects, including those related to the effects on quality of life, both behavioral outcomes, and finally economic/social issues, especially related to health care costs [9]. More precisely, clinical outcomes should include metabolic control and quality of life, and behavioral outcomes explore self-care and patient-caregiver interaction, while care coordination outcomes refer to cost-effectiveness, transparency of the care delivery process, equity of access to care, and usability of equipment to facilitate the care delivery process. The evaluation of the latter aspect, in particular, is quite missing in studies evaluating telemedicine in pregnancy complicated by diabetes (Tables 1 and 2).

### 2.1. Studies on Pregnant Women with Type 1 Diabetes

Wójcicki et al. [3] analyzed the effectiveness of an automated telematic intensive care system for transferring all of the patients' glucose measurements taken during the course of a day to a central clinical unit. The patients' mean blood glucose (MBG) and an indicator of glucose variability (the J index) were used to monitor their glycemic control. All the patients from both groups were initially educated during a 3-day program, which also included 2-day hospitalization. An additional training routine was performed during each patient's clinical visit for the entire duration of the study. Then patients were randomized to an experimental group, where the diabetologist performed an examination of the patient's state every day and had the opportunity to make changes to therapy, and a control group, where patients were treated only based on almost monthly clinical examinations without telephone contacts between patients and the diabetologist. The shortest period of application of the telematic system in telemedicine group was 137 days and the longest was 210 days (average, 180 ± 21.9 days), while in the control group the shortest time of application of the intensive treatment was 156 days and the longest was 208 days (average, 176 ± 16.4 days). The authors demonstrated a better glycemic control in the experimental group by comparison with a control group, based on the average differences in the patients' MBG and J indices, calculated weekly (ΔMBG = −3.2 ± 4.3 mg/dL, *P* = 0.0016, ΔJ = −1.4 ± 2.3, *P* = 0.0065). They also found a tendency for a better glycemic control in patients with lower intelligence quotient (IQ < 100) supported by the telematic system by comparison with all the other groups of patients, though this difference lacked statistical significance. The telematic intensive care system improved the efficacy of diabetes treatment during pregnancy.

Ładyżyński et al. [4] developed a system for supporting intensive insulin treatment in pregnant women with type 1 diabetes. The system consists of a patient teletransmission module (PTM) and a central clinical control unit (CCU). The PTM comprises a box containing a blood glucose meter and an electronic logbook, a modem for dial-up internet, or a cellular phone set. The CCU consists of a PC with a modem and DIAPRET software, a dedicated program designed to monitor the intensive insulin treatment. The system was tested on 15 pregnant type 1 diabetic women for 166 ± 24 days. Its total effectiveness was 69.3 ± 13.0% and its technical effectiveness was 91.5 ± 6.1%, and it was not significantly influenced by the patients' IQ, formal education, or place of residence, while it turned into a better metabolic control.

The same authors also assessed the influence of the greater frequency of data reporting on diabetic patients' metabolic control [5]. Data were reported via a home telecare system that stored blood glucose levels and was integrated with a simple electronic logbook. The data collected by patients were automatically transmitted via the telephone network every night. The study population consisted of 30 patients with type 1 diabetes, who were randomly allocated to the home telecare group or a control group. The control group's treatment was based on clinical examinations performed every three weeks. For the home telecare group, the data recorded by patients were transmitted to the hospital daily, enabling doctors to intervene more frequently. The average duration of the study was 180 days (SD 22) in the home telecare group and 176 days (SD 16) in the control group. The mean level of metabolic control and the insulin dose adjustment patterns were very similar in the two groups despite the much greater (15-fold) reporting frequency in the home telecare group. The data collected by patients were not fully usable, mainly because of the excessively high within-day variability in glycemic control and the high workload for the hospital staff performing the daily data analysis. On average, for the home telecare group, the patients' data were collected about 0.7 times per day (i.e., 15 times more often than in the case of routine treatment), although average metabolic control was found only slightly better for the home telecare group than for controls, and the number of adjustments to patients' insulin doses was very similar in the two groups. Both general compliance issues (relating to the considerable effort needed to analyze the daily data) and clinical problems (e.g., the high intraday glycemic variability) probably contributed to the lack of any significant differences between the two groups. These findings prompted the authors to conclude that remote systems used at home by patients with type 1 diabetes on intensive insulin therapy improve their glycemic control but need to support real-time data transmission and be combined with appropriate data analysis and subsequent decision-making for glycemic control to achieve any real improvement in the quality of care.

Di Biase et al. [6] also investigated whether telemedicine could be useful in the management of pregnant type 1 diabetic women. A fully automated system (the DIANET system) was used and 20 type 1 pregnant women took part in the study: 10 were treated using the telemedicine system and the other 10 using the conventional approach. The DIANET system was adopted at 4 different times, termed as “entry” (at 9.5 weeks of gestation), “basal” (9.5–16.8 weeks), “1st month” of investigation, and “end” (near delivery). All the women adopted intensified insulin administration protocols. DIANET group could record blood glucose values, insulin doses, diet and exercise information, ketonuria, and glycosuria; this system allowed patients to share these data with caregivers via modem-telephone; control group was not able to use DIANET system but was instructed, as well as DIANET group, to perform blood glucose tests 6-7 times/daily, namely, before and after meals and during night. Changes in therapy were performed every week for both groups. Judging from the profiles of the women's absolute blood glucose values, the DIANET ensured a better metabolic control than the conventional approach: values significantly lower before breakfast: 87 ± 6 versus 104 ± 4 mg/dL, lunch: 85 ± 5 versus 104 ± 4 mg/dL, and after dinner: 102 ± 5 versus 124 ± 6 mg/dL. These results were associated with higher insulin doses being used by the women in the DIANET group. There was significant reduction in both groups' hypoglycemic episodes at the “end,” “1st month,” and “basal” study points by comparison with the situation at “entry.” Based on their results, the authors suggest that telemedicine (DIANET) is a practical way to provide specialist care in pregnancy.

Frost and Beischer [7] used a remote data management system (CareLink; Abbott-MediSense, New Bedford, MA) to monitor 11 pregnant women with type 1 diabetes (all on intensive insulin therapy) from the 15th gestational week onwards, comparing them with controls receiving routine diabetes care, which consisted of visits every 2-3 weeks. The appointments made for the visits depended partially on the achieved metabolic control and the personal situation of the patients (e.g., distance between home and hospital and mothers with babies or young children). The authors used telemedicine system that allowed patients to transmit blood sugar levels from memory by the reflectometer (memory capacity up to 125 values) directly to a computer in their center through a modified modem over a telephone line. The controls were 10 pregnant women with type 1 diabetes matched for age, history of diabetes, and expertise with self-monitoring and insulin regimens. The average time between two visits was 3.3 weeks for the CareLink group and 2.9 weeks for the control group. There was an improvement in HbA1c in both the CareLink group (from 6.1 ± 1.0 to 5.4 ± 0.3) and the control group (from 6.2 ± 0.8 to 5.7 ± 0.6), though the differences were not statistically significant. MBG levels dropped in the CareLink group from 141 ± 90 to 110 ± 18 mg/dL and fasting glucose from 111 ± 17 to 101 ± 23 mg/dL (*P* < 0.05). Glycemic variability was also significantly reduced in both groups: the standard deviation of the MBG levels in individual patients fell from 51.6 to 44.4 mg/dL (*P* < 0.01), while for mean fasting blood glucose the SD decreased from 41.4 to 31.0 mg/dL. There was no significant reduction in the number of hypoglycemic episodes in either of the groups. The authors concluded that the system was easy to use and helpful in the treatment of diabetic women during pregnancy, enabling fewer outpatient visits. This aid would therefore be particularly suitable for women who have difficulty attending the prescribed regular check-ups at the clinic.

### 2.2. Studies on Pregnant Women with GDM

Dalfrà et al. [8] enrolled a total of 235 pregnant women (203 with GDM and 32 with type 1 diabetes mellitus) and assigned them sequentially to a telemedicine or a control group. Women with type 1 diabetes were enrolled in the study immediately after conception, while women with GDM were included one week after their GDM was diagnosed (at a mean 28 ± 1 weeks of gestation). The patients were sequentially assigned to two groups: one patient was followed up using the telemedicine approach and the next using the conventional approach. Women in telemedicine groups were trained on the use of the Glucobeep, monitoring their blood glucose levels with a glucometer (One Touch Ultra-Lifescan), and were asked to submit their glycemic data once a week, or more often if necessary, while they had a medical check-up at the diabetes clinic once a month. Women in control groups had a medical check-up every two weeks. All patients could contact the physician whenever they wished. Clinical and nonclinical outcomes were evaluated: the former included mode and timing of delivery, macrosomia, and maternal and fetal morbidity; the latter were deduced using questionnaires, that is, the CES-D for depression, the SF-36 for health-related quality of life (QoL), and the Stress and Distress on the impact of diabetes. The telemedicine GDM group achieved a better metabolic control in the third trimester (*P* = 0.008) and a lower rate of cesarean sections (*P* = 0.02) and macrosomia (*P* = n.s.). The women in the telemedicine group also had lower levels of frustration and concern about their diabetes and better acceptance of their diabetic condition. A strength of this study lies in that the authors adopted a straightforward telemedicine system (using the telephone) that was easy for all patients to handle, demanding no IT expertise or computer literacy.

Pérez-Ferre et al. [13] studied 97 women with GDM to ascertain the feasibility of a telemedicine system based on the Internet and text messaging and its influence on delivery and neonatal outcomes (HbA1c values <5.8%, normal vaginal deliveries, and LGA babies). All women were instructed as regards nutritional habits and self-monitoring of capillary blood glucose; one week later values of glucose monitoring were evaluated and patients were randomised 1 : 1 in two groups (control and telemedicine), according to age and obstetric history. Forty-eight women attended traditional face-to-face visits and were treated in accordance with standard face-to-face monitoring outpatient protocol, and 49 women formed the experimental group using the telemedicine system to send capillary glucose data and short text messages, receiving professional feedback weekly. Women in the control group were followed up with blood glucose targets and could attend the outpatient clinic without prior appointment (nonscheduled visit) and could ask for their blood glucose values or any queries regarding nutritional recommendations or insulin dose. Conversely, women in the intervention group received a glucometer with a cellular phone, with the possibility of transmitting capillary glucose values to a central database, via short message service, and also contacting health professionals when required. Caregivers had full access to patients' data, analyzing glycemic trends over time, charts of everyday and weekly glucose values, and the daily glycemic values of every patient. Then, health professionals could make recommendations for changes to nutritional regimens and/or insulin doses.

There was no significant difference between the two groups in terms of the outcomes considered, despite the experimental group's significantly reduced number of visits to the clinic (0.38 ± 0.68 versus 1 ± 1.35 per patient; *P* < 0.03), particularly among the insulin-treated women (0.50 ± 0.73 versus 2.89 ± 1.05 per patient; *P* < 0.001), compared with conventional monitoring. The authors concluded that the telemedicine-based system achieved similar pregnancy, delivery, and newborn outcomes to the traditional treatment approach, while significantly reducing the need for outpatient clinic visits.

More recently [14], the same authors demonstrated that, compared with a control group, a telemedicine group reduced the number of unscheduled face-to-face visits by 62% (and by 82.7% for the subgroup of insulin-treated patients), improving patient satisfaction and achieving comparable pregnancy and newborn outcomes.

In a study by Homko et al. [15], women with GDM were randomized to either an Internet group (*n* = 32) or a control group (*n* = 25). Women in both groups were asked to monitor their blood glucose levels daily (before breakfast and 2 h after each meal), perform fetal movement counting three times a day, and also record insulin doses and episodes of hypoglycemia. Women in the treatment group were asked to transmit this information via either the phone or the Internet at least weekly to their healthcare providers. Women in the control group were asked to record this information in a logbook, which was reviewed by the medical team at prenatal visits. Patients in the Internet group were given computers and/or Internet access as necessary. A website was established for recording glucose levels and for communications between patients and the health care team. Women in the control group kept paper logbooks, which were reviewed at each prenatal visit. Maternal feelings about diabetes self-efficacy were assessed at study entry and again before delivery. Women in the Internet group accessed the system and sent a mean 21.8 (±16.9) sets of data. There was no difference between the two groups' fasting or postprandial blood glucose levels, although more women in the Internet group were on insulin therapy (31% versus 4%; *P* < 0.05). There were also no significant differences in pregnancy and neonatal outcomes between the two groups. The women in the Internet group demonstrated a significantly stronger sense of self-efficacy at the end of the study. The potential benefits of monitoring blood glucose via the Internet in indigent women with GDM were limited by their infrequent use of the telemedicine system. While using the system was not associated with better pregnancy outcomes, the diabetic women in the telemedicine group did experience a better sense of psychosocial self-efficacy.

In a subsequent study [16], these authors tested a more advanced telemedicine system, which included automated reminders to patients to send their data. Eighty GDM women were randomized to join an intervention group using telemedicine to send blood glucose recordings obtained 4 times a day via the Internet or telephone, or a control group using paper logbooks. Although there were no significant differences in the outcomes considered (glucose control and birth weight of offspring), this type of telemedicine approach improved the contact between patients and healthcare professionals, making the use of technology for monitoring of diabetes in pregnancy more familiar.

Finally, in GDM patients one study showed that integrating telemedicine applications and involvement of the nursing staff turns into better fetal outcome and adhesion to glucose monitoring. With this respect Ferrara et al. demonstrated that higher referral frequency to telephonic nurse management for gestational diabetes mellitus decreased risk of macrosomic infant and increased postpartum glucose testing [19]. Their nurse-based management program offered supplemental care via telephone counseling to women with high risk pregnancies, including a call center with 32 registered nurses and 2 registered dietitians. Telephone counseling was guaranteed 7 days a week on glucose monitoring and control, diet, and physical activity. The center has provided up to 2 extra calls per week, in addition to the care provided by obstetricians, to help pregnant women in the management of blood sugar levels.

## 3. Effectiveness for Maternal-Fetal Outcomes and Cost-Benefit Assessment: Key Issues for the Future

The maternal complications associated with diabetes in pregnancy are gestational hypertension, higher rates of cesarean section and preeclampsia, and a higher risk of developing type 2 diabetes, especially after menopause. As for the adverse fetal outcomes, fetal hyperinsulinemia is a pathophysiological trigger that through the overgrowth of insulin-sensitive tissues (especially adipose tissue) turns maternal hyperglycemia into unbalanced fetal growth and a consequent increased risk of trauma at birth, shoulder dystocia, and perinatal death. Hyperinsulinemia is also responsible for many neonatal metabolic complications, including hypoglycemia, hyperbilirubinemia, hypocalcemia, hypomagnesemia, polycythemia, respiratory distress syndrome, and a higher long-term risk of diabetes mellitus and childhood obesity. Pregnancy complicated by obesity is characterized by high rates of adverse maternal and fetal outcome too, especially but not only in patients with GDM [20]. Some of the studies included in this review did not consider the pregestational weight [3, 6, 7], while others have specified both the body mass index (BMI) and weight gain during pregnancy [8, 13–16]. In general, there were no significant differences in these parameters between intervention groups and control groups. However, in many studies the distribution range of BMI and weight gain was particularly wide. Therefore, it may be important to evaluate subgroups of patients with more restricted BMI, so as to verify its relationship with the therapeutic intervention. Providing adequate information for women at risk and ensuring that they attend the clinic regularly are pivotal strategies for reducing the rate of these complications.

It follows that the ideal approach to treating diabetes in pregnancy necessarily includes frequent blood glucose self-monitoring, adequate dietary guidelines that take the mother's initial weight and the various stages of gestation into account, and appropriate insulin therapy where necessary [21]. It is striking how much telemedicine can help in the management of these patients by enabling the above-mentioned clinical needs to be addressed.

Unfortunately, it is clear from the data summarized in the previous paragraphs that, although the effects of telemedicine on glycemic control and psychological aspects related to diabetes in pregnancy have been confirmed, its efficacy in improving maternal and fetal outcomes remains to be demonstrated. It is also still not clear whether telemedicine is really more advantageous, in cost-benefit terms, than traditional clinical management.

For both of these aspects, it will be especially important to plan prolonged clinical trials (over years rather than months) and assess quantitative indices from which pooled estimates of effect can be calculated. For example, these indices could include quality of life, measured using appropriate scales depending on the diseases considered, or emergency department visits, or number of days in hospital [22]. It would also be useful if the outcomes considered were standardized and were identical for all intervention studies. Finally, only trials on a sizable number of subjects should be considered valid (much of the currently available evidence is based on samples of a few dozen patients).

## 4. Conclusion

**Figure 1:**
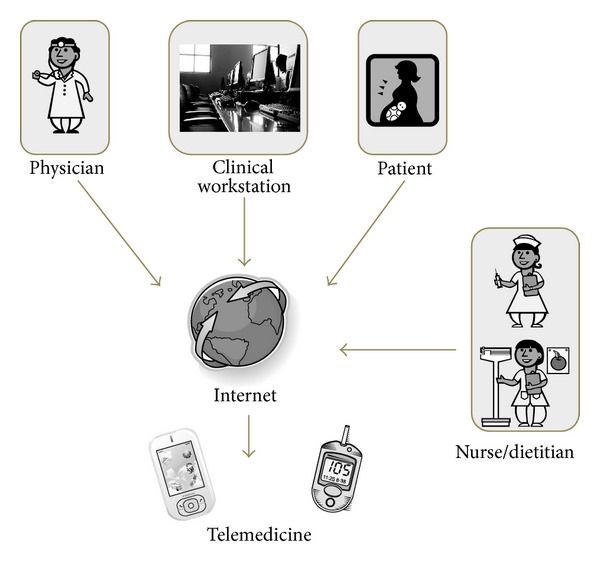
Schematic representation of the interconnection between patients, specialists, and technologies supported by telemedicine.

Various aspects of telemedicine are emerging as being of interest for managing diabetes in pregnancy. Implementing telemedicine in daily clinical practice can be just as effective as conventional clinical management for the purposes of glycemic control and maternal-fetal outcomes and even superior in terms of the impact on patients' quality of life and the cost-benefit ratio. In particular, telemedicine can cope with the needs of more and more patients, as the time available for visits becomes less and less. With fewer visits to the doctor, costs can be cut while retaining the same level of care (even after the costs of creating a telemedicine system have been taken into account). Implementing telemedicine in the clinical management of diabetes in pregnancy also facilitates greater involvement of other professionals, such as nurses and dietitians (Figure 1), whose support can help save time and resources in the follow-up of these patients [23]. Telemedicine definitely has the potential to revolutionize current methods for managing pregnancy complicated by diabetes, bringing benefits to both patients and health care systems [24].

## Figures and Tables

**Table 1:** Brief summary of the main outcomes of the studies conducted in pregnant women with type 1 diabetes.

	Wójcicki et al. [3]	Ładyżyński et al. [4]	Ładyżyński and Wójcicki [5]	Di Biase et al. [6]	Frost and Beischer [7]	Dalfrà et al. [8]
Number of participants (interv./control)	15/15	15/nv	15/15	10/10	11/10	17/15
Duration of monitoring (days ± SD; interv./control)	180 ± 21.9/176 ± 16.4	166 ± 24	180 ± 22/176 ± 16.4	Variable between groups	158 ± 41/188 ± 32	167 ± 27/160 ± 63
Subjects training (interv./control)	Telematic ICS/RAV	PTM and CCU/nv	HTS/CE every 3 weeks	DIANET system/RAV	CareLink system/RAV	Glucobeep system/CE every 2 weeks
Clinical outcome (metabolic/QoL)	+/nv	+/nv	=/+	+/nv	+/nv	=/+
Behavioural outcome	nv	+	nv	nv	nv	+
Care coordination outcome	nv	nv	nv	nv	nv	nv

For more details about the clinical, behavioral, and care coordination outcomes, refer to Verhoeven et al. [9].

QoL: quality of life; SD: standard deviation; nv: not valued; ICS: intensive care system; RAV: regular ambulatory visits; PTM: patient teletransmission module; CCU: central clinical control unit; HTS: home telecare system; CE: clinical examinations.

**Table 2:** Brief summary of the main outcomes of the studies conducted in pregnant women with GDM.

	Pérez-Ferre et al. [13]	Pérez-Ferre et al. [14]	Homko et al. [15]	Homko et al. [16]	Dalfrà et al. [8]
Number of participants (interv./control)	49/48	49/48	32/25	40/40	88/115
Duration of monitoring (days ± SD; interv./control)	94 ± 26/94 ± 24	94 ± 26/94 ± 24	71 ± 40/69 ± 38	71 ± 39/67 ± 38	76 ± 25/75 ± 27
Subjects training (interv./control)	Internet and SMS/TSV	Internet and SMS/TSV	Website for recording glucose values/paper logbook	Website for recording glucose values/paper logbook	Glucobeep system/CE every 2 weeks
Clinical outcome (metabolic/QoL)	=/nv	=/+	=/+	=/nv	+/+
Behavioural outcome	nv	+	+	+	+
Care coordination outcome	+	+	nv	nv	nv

For more details about the clinical, behavioral, and care coordination outcomes, refer to Verhoeven et al. [9].

QoL: quality of life; SD: standard deviation; nv: not valued; SMS: short message service; TSV: traditional face-to-face visits; CE: clinical examinations.
